# Function of membrane domains in rho-of-plant signaling

**DOI:** 10.1093/plphys/kiaa082

**Published:** 2021-01-05

**Authors:** Marija Smokvarska, Yvon Jaillais, Alexandre Martinière

**Affiliations:** 1 BPMP, CNRS, INRAE, Univ Montpellier, Montpellier SupAgro, 34060 Montpellier, France; 2 Laboratoire Reproduction et Développement des Plantes, CNRS, INRAE, Université de Lyon, ENS de Lyon, UCB Lyon 1, F-69342 Lyon, France

## Abstract

In a crowded environment, establishing interactions between different molecular partners can take a long time. Biological membranes have solved this issue, as they simultaneously are fluid and possess compartmentalized domains. This nanoscale organization of the membrane is often based on weak, local, and multivalent interactions between lipids and proteins. However, from local interactions at the nanoscale, different functional properties emerge at the higher scale, and these are critical to regulate and integrate cellular signaling. Rho of Plant (ROP) proteins are small guanosine triphosphate hydrolase enzymes (GTPases) involved in hormonal, biotic, and abiotic signaling, as well as fundamental cell biological properties such as polarity, vesicular trafficking, and cytoskeleton dynamics. Association with the membrane is essential for ROP function, as well as their precise targeting within micrometer-sized polar domains (i.e. microdomains) and nanometer-sized clusters (i.e. nanodomains). Here, we review our current knowledge about the formation and the maintenance of the ROP domains in membranes. Furthermore, we propose a model for ROP membrane targeting and discuss how the nanoscale organization of ROPs in membranes could determine signaling parameters like signal specificity, amplification, and integration.

## Teaser

The nanoscale organization of Rho of Plant proteins creates emergent properties that determine cellular signaling.

## Introduction

**Figure 1:**
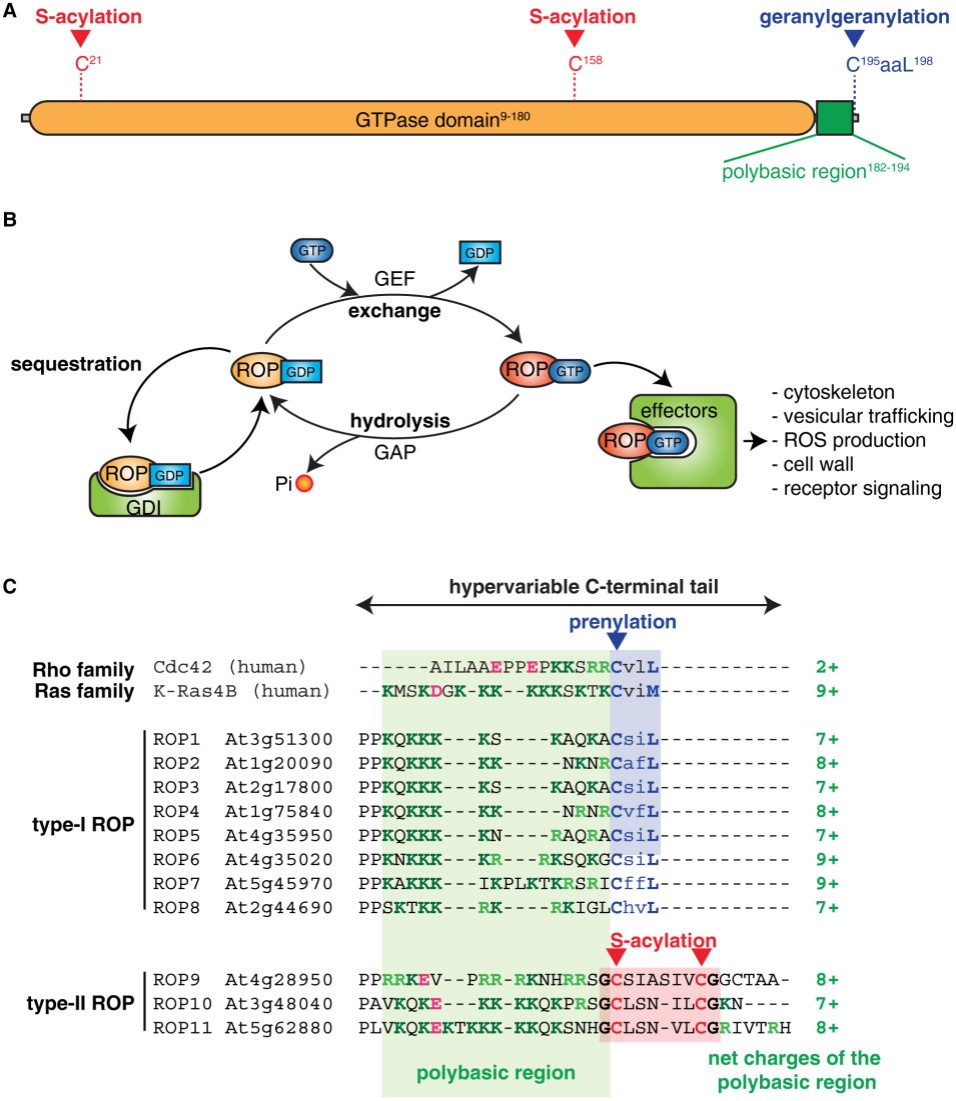
ROP structure and GTPase cycle. A, Schematic representation of the linear architecture of type-I ROPs. The residue number (in superscript) is given for ROP6 as an example. B, Schematic representation of the ROP GTPase cycle. C, Alignment of the C-terminal hypervariable region of the 11 Arabidopsis ROPs, showing the differences between type-I and type-II ROPs. Cationic residues are highlighted in green, acidic residues are highlighted in pink, the CaaX prenylation motif is in blue (CaaL: geranylgeranylation, CaaM: farnesylation) and the GC–CG *S*-acylation motif in red. For comparison purposes, the sequence of the C-terminal tail of human Cdc42 and K-Ras4B is included, as the archetypal representative of the Rho and Ras family, respectively. GEF, GTPase Exchange Factor; Pi, inorganic phosphate.

Rho of Plant (ROP) proteins are members of the small GTPase family that are about 20 kDa, essentially made of their GTPase domain (also called a G domain) with short N- and C-terminal extensions (Figure 1, A). ROP proteins are defined by their basic biochemical activity of binding guanosine triphosphate (GTP) and hydrolyzing it into guanosine diphosphate (GDP), also known as GTP/GDP cycle (Figure 1, B; Bourne et al., 1991). Classically, the GDP-bound form is inactive, while the GTP-bound form is active and can associate and activate downstream proteins (hereafter referred to as “effectors”; Figure 1, B). This process represents a ubiquitous regulatory mechanism in cells, making small GTPase molecules behave like a digital switch (Henis et al., 2009). The control of these switches comes via the association of GTPases with additional proteins. First, guanine nucleotide exchange factors (GEFs) catalyze the conversion of small GTPase from their GDP-bound state into their GTP-bound state, therefore, placing them in their active conformation (Figure 1, B). GEFs also facilitate the release of the small GTPase activating proteins (GAPs) that increase GTP hydrolysis and serve as small GTPase inhibitors (Figure 1, B; Berken and Wittinghofer, 2008). A third regulatory element of some small GTPases is the guanosine nucleotide dissociation inhibitors (GDIs) which prevent activation by keeping GDP-bound GTPases from localizing to membranes (Figure 1, B; Cherfils and Zeghouf, 2013).

**Advances**

- Rho of Plant (ROP) proteins regulate cell polarity and are themselves polarly localized, often via self-organizing systems involving guanine nucleotide exchange factors and GTPase activating proteins.
- The organization and dynamics of ROP membrane localization at the nanoscale are pivotal for ROP function.
- Lipid modifications and lipid interactions are involved in ROP polarity, nanodomain formation, and function.
- Anionic lipids act as a signaling rheostat that modulates signaling output during development.
- The input-specific composition of ROP nanodomains contributes to output specificity.

The small GTPase family is one of the largest families of signaling molecules in eukaryotes. It is divided into five distinct families: Ras, Rab, Arf, Ran, and Rho (Kahn et al., 1992). Members of the Rab and Arf families mostly function in the regulation of vesicular trafficking, including the formation of vesicles and tubules, regulation of organelle dynamics via interaction with cytoskeleton components, vesicle docking on acceptor membranes, and the specification of membrane identity or compartment maturation (Gillingham and Munro, 2007). Ras-related nuclear proteins (Ran) on the other hand regulate the transport of proteins and RNA across the nuclear envelope (Kalab et al., 2002). Aside from nucleocytoplasmic transport, Ran has also been implicated in a variety of other cellular functions such as proper mitotic spindle assembly for chromosome alignment during mitosis (Clarke and Zhang, 2008), and increased apoptosis and suppression of cell proliferation in human cells (Deng et al., 2013). The two last families of eukaryotic small GTPases are often grouped in the so-called Ras superfamily and are involved in cell surface signaling. Members of the Ras subfamily are well-described regulators of cell proliferation downstream of growth factor signaling, while members of the Rho subfamily are mainly involved in the regulation of cytoskeleton dynamics, especially actin filaments.

Plants lack the Ras subfamily, hence ROPs is the sole representative of the Ras superfamily. However, to some extent, ROPs can fulfill functions that are attributed to either Rho and/or Ras in animals. Like canonical animal Rho GTPases, ROPs are involved in signaling events that regulate cytoskeletal organization and vesicular trafficking and thus, impact on cell polarization and polar growth (Kawasaki et al., 2006). In addition, ROPs also relay and integrate signals downstream of receptor kinases, which is typically the function of Ras GTPases in animals. At the structural level, ROPs contain elements of Rho and Ras proteins. Indeed, their GTPase domain is closely related to that of Rho GTPases. This explains that plants and animals Rho share effectors with common domains, such as the Cdc42 and Rac-interactive binding (CRIB) domain. However, the ROP hypervariable C-terminal tail is more related to that of the archetypal Ras protein K-Ras4B than to the Rho protein Cdc42 (see Figure 1, C). Given the importance of this region in small GTPase localization (see below), this difference has a substantial impact on ROP membrane dynamics, which by many accounts are closer to Ras than Rho proteins. Thus, throughout this review, and when appropriate to discussing their function and regulation, we will draw parallels between ROPs and animal Rho, and also include Ras proteins.

Since the fluid mosaic model was proposed in the early 70s, our vision of membrane organization has become more complex (Martinière and Runions, 2013; Jaillais and Ott, 2020). Substantial studies involving advanced microscopy techniques revealed that the plasma membrane contains different domains with different biophysical properties wherein proteins and lipids involved in signaling can selectively interact with their effector molecules. The distribution of small GTPases into organized membrane domains is essential for their regulation, especially in the case of the Rho and Ras subfamilies. In plants, the understanding of the impact of membrane domain formation on ROP activity is also increasing. Here, we will review the recent advances in our understanding of how membrane domains, from the micrometer to nanometer size, can influence ROP signaling in plants, and to what extent ROP spatio-temporal regulation acts in ways similar or different to other eukaryotic systems.

## ROP membrane domains

ROPs segregate laterally at the plasma membrane in membrane domains. These domains vary in size and, according to the nomenclature introduced by Ott (2017), they are referred to as microdomains when bigger than 1 µm and nanodomains when they are below that limit (Ott, 2017).

### ROP membrane microdomains

A considerable number of ROP isoforms can form microdomains within the membrane. These structures are bigger than 1 µm, relatively stable over time, and are hallmarks of cell polarity. Therefore, they can be easily visualized with conventional fluorescence microscopy (Ott, 2017). Among some of the first polarity markers found are the auxin efflux carriers from the PIN family (Gälweiler et al., 1998). After that, a great number of proteins and lipids were found in membrane microdomains, including the four sides and corners of root cells (i.e. rootward, shootward, inner and outer lateral polar domains), plasmodesmata, lobe and neck regions of leaf pavement cells, plant–microbe interfaces, the different regions of tip growing cells (i.e. shank, sup-apical, and apical), or the sites of local cell wall modification (e.g. Casparian strip, pollen aperture, xylem pit-field, trichome; Tapken and Murphy, 2015; Barbosa et al., 2019; Sapala et al., 2019; Sauer and Kleine-Vehn, 2019; Yoshida et al., 2019; Bozkurt and Kamoun, 2020).

#### ROP polarity in tip growing cells

There are two cell types in plants that grow using tip growth: pollen tubes and root hairs. Tip growing cells are highly polarized, with distinct polar domains corresponding to the very tip (or apical part of the growing cells), the sub-apical region, and the shank of the tube/hair. Over the years, ROPs emerged as master regulators of polarized tip growth in plants, in both root hairs and pollen tubes.

**Figure 2:**
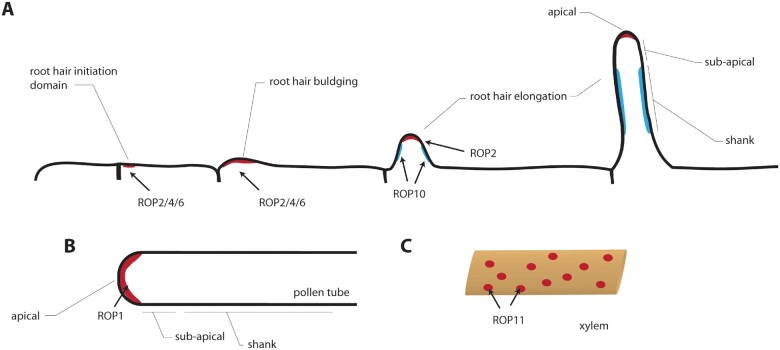
ROP microdomains in different cell types. A, In a trichoblast cell, ROP2/4/6 accumulate in the root hair initiation domain (RHID). During hair elongation, ROP2 (red) is present at the root hair tip whereas ROP10 (blue) accumulates at the shank. B, In the pollen tube tip, ROP1 is present in a microdomain. Its accumulation fluctuates over time during pollen tube growth. C, During xylem differentiation, ROP11 is present in microdomains that lead to the formation of cell wall pits.

In Arabidopsis (*Arabidopsis thaliana*), ROP2, ROP4, and ROP6 localize in a large microdomain (around 5–10 µm) at the root hair initiation domain (Molendijk et al., 2001; Jones et al., 2002; Fischer et al., 2006; Ikeda et al., 2009; Denninger et al., 2019; Figure 2, A). This domain marks the future site where the root hair will initiate in root hair-bearing epidermal cells (i.e. trichoblasts). Importantly, root hairs initiate at a constant and predictable position along the trichoblast: next to, but not directly at, the rootward pole of the cell (Masucci and Schiefelbein, 1994; Grebe et al., 2002; Stanislas and Jaillais, 2019). ROP recruitment at the root hair initiation domain follows a two-step assembly: first, an initiation phase where the root hair initiation domain is predefined by GEF3 and drives ROP2/4/6 localization; then a polar growth phase, that is sustained by GEF4 (Denninger et al., 2019; Stanislas and Jaillais, 2019). The roles of the different ROP isoforms were also illustrated by the use of constitutively active (CA, constitutively locked in the GTP-bound conformation) or dominant-negative (DN, locked in a GDP-bound conformation) ROPs. For example, both ROP4-CA and ROP6-CA expression induces root hair swelling (Molendijk et al., 2001), and ROP2-DN results in less and shorter hairs, whereas ROP2-CA plants produce more and longer hairs than wild type (Jones et al., 2002). The same was confirmed with loss of function single, double, or triple mutants for *rop2*, *rop4*, *rop6*. Hair length was reduced by about 70% in the *rop2 rop4RNAi rop6* triple line (Gendre et al., 2019).

While GEF3 is required to trigger ROP localization in the root hair initiation domain, GAPs are involved in fixing this polarity. Indeed, ARMADILLO REPAT ONLY (ARO) proteins (ARO2, ARO3, and ARO4) are scaffolding proteins that directly interact with the ROP1 ENHANCER (REN1–3), also known as PHGAP proteins (Hwang et al., 2010; Stöckle et al., 2016), GAP and activated GTP-loaded ROP (Kulich et al., 2020). In the *aro2 aro3 aro4* triple mutant, ROP2 initially accumulates at the root hair initiation domain, but fails to maintain this localization. REN1 and ROP2 localize in complementary domains at the plasma membrane of nascent root hairs, in the shank and tip, respectively. However, REN1 and ROP2 also colocalize at the junction between the two root hair domains (Kulich et al., 2020). This suggests that REN proteins inactivate ROP2 when it leaks outside of its polar domain. Because AROs are required for the plasma membrane localization of REN proteins, this model could explain how RENs/AROs fix the polar localization of ROP2. Together, these studies nicely illustrate how concerted GEF and GAP activities can be used to properly control ROP polarity (Denninger et al., 2019; Stanislas and Jaillais, 2019; Kulich et al., 2020).

In addition to GEF/GAP, the trans-Golgi network (TGN)-localized YPT-INTERACTING PROTEIN (YIP) 4a and 4b are central trafficking components in ROP activation and ROP-guided root hair initiation (Gendre et al., 2019). Indeed, the secretion-defective *yip4a yip4b* mutant has decreased levels of ROP2 at the root hair initiation domain, which likely leads to shorter root hairs (Gendre et al., 2019). Gendre et al. (2019) proposed that ROP2 can be found in the TGN (e.g. SYP61-containing vesicles), which are part of the secretory pathway and could be essential for ROP activation and polar accumulation.

The importance of the proteo-lipid environment in the establishment of the root hair initiation domain was exemplified by Stanislas et al. (2015). They described that ROP2 and ROP6 accumulate together with phosphatidylinositol-4-phosphate 5-kinase 3 (PIP5K3), DYNAMIN-RELATED PROTEIN (DRP) 1A and 2B in a sterol-rich membrane domain. They uncovered that ROPs, PIP5K3, and DRPs are localized to the root hair initiation domain before root hair bulging. By contrast, D6 PROTEIN KINASE (D6PK), which serves as a modulator of this process, switches from a rootward polarity to the root hair initiation domain at the time of root hair emergence (Stanislas et al., 2015).

The cytoskeleton can also participate in ROP membrane localization. Indeed, MICROTUBULE-ASSOCIATED PROTEIN 18 (MAP18) was shown to interact genetically and physically with ROP2, controlling ROP2 localization at the root hair initiation domain (Kang et al., 2017). Moreover, the maintenance of polarized ROP in the root hair initiation domain is related to its ability to be dissociated from the membrane by GDI activity. The absence of SCN1/RHOGDI1 induces the formation of an ectopic GFP-ROP2 microdomain, leading to super numerate root hair initiation domains and eventually the formation of several root hairs per trichoblast (Carol et al., 2005). Oppositely, SCN1/RHOGDI1 overexpression dislodges ROP2-CA from the membrane in guard cells (Jeon et al., 2008). Later when the root hair starts to elongate, ROP10 gets recruited to the cell shank. This atypical localization is mediated by a phosphoinositide kinases FORMATION OF APLOID AND BINUCLEATE CELLS 1 (FAB1) or its direct product phosphatidylinositol-3,5-bisphosphate (PI(3,5)P_2_; Hirano et al., 2018). Together, they coordinate microtubule organization and deposition of cell wall components that contribute to the stiffness of the root hair shanks (Hirano et al., 2018).

The link between the regulation of the ROP cycle and its polarized localization in tip growing cells was also explored in the pollen tube model system (Qin and Yang, 2011). In this system, Arabidopsis ROP1, and to a lesser extent ROP5, as well as *Nicotiana tabacum* NtRac5, forms a tip localized domain (Gu et al., 2005; Klahre et al., 2006; Klahre and Kost, 2006; Ischebeck et al., 2011; Stephan et al., 2014; Sun et al., 2015; Feng et al., 2016; Figure 2, B). Their maintenance at the tip is determined by several GAP proteins. First, ROP1 ENHANCER 1 (REN1) is localized in subapical cytoplasmic vesicles and to the apical membrane (Hwang et al., 2010). As *ren1* has a swollen pollen tube and enlarged ROP1 domain, it is thought to control ROP1 polar localization through a negative feedback-based mechanism (Hwang et al., 2010).

More recently, another GAP, REN4 was associated with this interplay (Li et al., 2018). REN4 is mostly localized on the lateral plasma membrane region, but shows a transient tip localization. When REN4 accumulates in the pollen tube tip, the intensity of fluorescently tagged ROP1 decreases. REN4 is controlling ROP1 membrane association by initiating its removal from the plasma membrane through clathrin-mediated endocytosis. In addition, the temporal oscillation of REN1 and REN4 at the pollen tube tip spatially controls ROP1 localization and consequently participates in pollen tube growth (Qin and Yang, 2011; Li et al., 2018).

The importance of ROP negative regulators is also supported from studies in *N. tabacum* pollen tubes, with NtRhoGDI1 and NtRhoGAP1 both being involved in the precise spatio-temporal regulation of NtRac5 close to, but not in, pollen tube tips (Klahre et al., 2006; Klahre and Kost, 2006; Sun et al., 2015). Restriction of Rho GTPase signaling to the growth site by negative regulators appears to be a common mechanism between different organisms. For example, GAP loss-of-function or expression of CA Cdc42 GTPase leads to increased growth depolarization in filamentous fungi (Ushinsky et al., 2002).

#### ROP microdomains in cell wall patterning

In metaxylem cells, cell wall pits are formed thanks to GTP-loaded ROP11 organized in microdomains of tens of micrometers (Figure 2, C). The maintenance of those microdomains requires the coordination between the ROP11 activator, GEF4, and the ROP11 inhibitor, GAP3 (Oda and Fukuda, 2012; Nagashima et al., 2018). Interestingly, the reconstruction of evenly distributed domains can be achieved in a heterologous system, solely using ectopic expression of the three proteins (Oda et al., 2018). This reconstruction requires intact ROP11 that can cycle between its GTP-bound and GDP-bound states, as GTP-locked ROP11 induced disorganization of GEF4 in membranes. In addition, ROP11 microdomains are spatially restrained by cortical microtubules through the IQ67-DOMAIN13 (IQD13) and CORTICAL MICROTUBLE DISORDERING1 (CORD1) proteins (Sasaki et al., 2017; Sugiyama et al., 2017). The ROP11 effector MICROTUBULE DEPLETION DOMAIN1 (MIDD1) is recruited within ROP11 microdomains and promotes microtubule depolymerization and inhibition of cell wall deposition in the pit area by interacting with the kinesin KIN13A (Oda and Fukuda, 2012; Oda and Fukuda, 2013). More recently, a second protein complex including BOUNDARY OF ROP DOMAIN1 (BDR), a ROP11 effector, and WALLIN (WAL) was shown to promote actin polymerization. This complex stimulates cell wall deposition at the pit boundaries (Sugiyama et al., 2019). The coordination, including of components of the cytoskeleton like actin and microtubules, between these two signaling pathways, with opposite effects on the cell wall growth, can ensure the establishment of specialized cell wall domains.

#### ROP microdomains in pavement cell shaping

The jigsaw puzzle shape of pavement cells in the leaf epidermis serves as an exciting model to investigate the mechanisms for cell shape formation (Wasteneys and Galway, 2003). The development of Arabidopsis pavement cells is divided into three stages: Stage I starts when pentagonal or hexagonal initial cells expand along the leaf long axis to form slightly elongated polygons. These cells initiate multiple outgrowths or localized lateral expansion from their anticlinal walls into adjacent cells, producing stage II cells with multiple shallow lobes alternating with indentations or necks. As early lobes expand, reiterative lobe and neck formation continues, resulting in highly lobed interlocking cells (stage III). The cell-to-cell signaling is crucial for spatiotemporal coordination of lobe outgrowth with inhibition of outgrowth in the corresponding indented region of the adjacent cell (Fu et al., 2005). The cytoskeleton is also implicated in pavement cell development. Cortical microtubule bundles arranged transversely in the neck regions and can restrict expansion (Wasteneys and Galway, 2003). In contrast, lobe initiation and outgrowth appear to require cortical fine actin filaments localized to sites lacking well-ordered cortical microtubules (Frank and Smith, 2002; Fu et al., 2002). ROP2 and ROP6 have been described to fine-tune different parts of the cytoskeleton to achieve the jigsaw puzzle shaping of the pavement cells. ROP2 promotes the formation of cortical diffuse F-actin and lobe outgrowth via its effector ROP-INTERACTIVE CRIB MOTIF-CONTAINING PROTEIN4 (RIC4; Fu et al., 2005). In the lobe, ROP2 suppresses well-ordered cortical microtubules by inactivating another effector, RIC1 (Fu et al., 2002, 2005). In the opposite neck region, ROP6 activates RIC1 to promote well-ordered microtubules and to suppress ROP2 activation (Fu et al., 2005, 2009). In *N. tabacum*, NtRAC1, a type-I ROP, is activated by auxin (Tao et al., 2002; Wu et al., 2011). Similar assays later showed that Arabidopsis ROP2 and ROP6 are also activated by auxin (Xu et al., 2010), and that this activation is dependent on the TRANSMEMBRANE KINASE (TMK1–4) leucine rich repeat receptor-like kinase (LRR-RLK) pathway (Xu et al., 2014). Auxin orchestrates the polarization of PIN1 that together with ROP2 and their positive feedback loop acts with the antagonizing ROP6 pathway to generate localized extracellular (or apoplastic) auxin. The activation of ROP2 and ROP6 pathways by the TMK receptors could explain how uniform concentrations of auxin lead to the establishment of cell regions that define lobe or indentation forming sites (Xu et al., 2010, 2014). Note that Xu et al. (2010, 2014) reported that the AUXIN BINDING PROTEIN1 (ABP1) acts upstream of TMK1 and ROPs in the perception of the auxin signal. However, this finding has been called into question as the *abp1* alleles used in these studies were not targeting the *ABP1* gene and true *ABP1* loss-of-function have no detectable phenotypes (Dai et al., 2015; Enders et al., 2015; Gao et al., 2015; Michalko et al., 2015, 2016).

### ROP membrane nanodomains

Technological advances within the field of microscopy, including total internal reflection fluorescence microscopy (TIRFM) and super-resolution microscopy applied to plant samples have enabled more precise definition of protein organization in membranes (Konopka and Bednarek, 2008; Kleine-Vehn et al., 2011; Li et al., 2011, 2012; Martinière et al., 2012; Hao et al., 2014; Hosy et al., 2015). Because of the development of these powerful microscopy techniques, an increasing number of molecules have been described to be localized in domains smaller than a micron. Nanodomain-localized proteins include proteins involved in various physiological and molecular contexts, for example in auxin transport (Kleine-Vehn et al., 2011), abscisic acid signaling (Demir et al., 2013), immunity (Bücherl et al., 2017; McKenna et al., 2019), and many more.

Recently, the role of protein nanodomain organization during cell stimulation was exemplified in the case of ROP signaling. Platre et al. (2019) showed that ROP6 is recruited in nanoclusters of 50–70 nm, only a few minutes after auxin stimuli in Arabidopsis root cells. Constitutive active ROP6 accumulates in nanodomains in the absence of any cell stimulation, suggesting that the ROP6 nanodomain concentrates GTP-bound ROP6. ROP6 also nanoclusters upon osmotic stimuli (Smokvarska et al., 2020). GTP-bound ROP6 is remobilized in clusters together with its effector RESPIRATORY BURST OXIDASE HOMOLOG (RBOH). This enzyme is responsible for ROP6-dependent ROS accumulation upon sorbitol treatment. Whereas ROP6 variants that cannot be redirected into clusters show no increase in ROS accumulation upon activation, ROP6-CA expressing plants display high ROS and numerous nanodomains without any cell stimulation. More importantly, the RBOH recruitment in nanodomains is constitutive in this condition, suggesting that activated ROP6 can meet RBOH in specific plasma membrane nanodomains (Smokvarska et al., 2020).

It seems that the nanoclustering of ROPs is a general feature for many developmental and adaptation processes. Clustering of active GTPase in nanoclusters is a shared feature in yeast and animals, including K-Ras, Rac1, and Cdc42 (Prior et al., 2003; Remorino et al., 2017; Meca et al., 2019). The mechanisms behind the formation of ROP-containing membrane domains are complex and regulated by many different factors in parallel, and thus remain partially understood. But some of the common underlying principles for the formation of polar domains and nanodomains are emerging in plants, including self-organization of the ROP/ROP-GEF/ROP-GAP module, lipid modifications, and lipid interactions (Nagashima et al., 2018; Platre et al., 2019).

## Domain formation and maintenance

### Domain self-organization by a reaction–diffusion system

**Figure 3:**
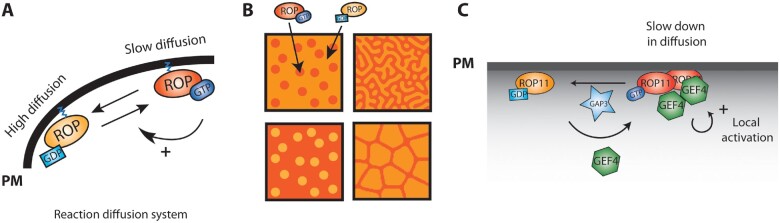
ROP self-organization through a reaction–diffusion system. A, Two chemical species (here ROP-GDP and ROP-GTP bound) are under a reaction–diffusion process when long-range inhibition by a higher diffusion of the inhibitor and local activation by a slower diffusion of the activator exist. B, Changing various parameters in the reaction–diffusion equation leads to an array of two dimension patterns. C, In the case of cell wall pit formation, ROP11 is globally inhibited by the cytoplasmic GAP3. It gets activated by GEF4, which decreases its diffusion. As GEF4 makes dimers, it induces a local recruitment of new ROP11 molecules.

Recent studies proposed a novel ROP membrane domain organization via self-organization processes, where overall order arises from local interaction between components of a disordered system (Figure 3, A). Activator–inhibitor systems are reaction–diffusion processes, which are based on two chemical species where one is a local activator with a slow diffusion and the other one creates a global inhibition from its fast diffusion (Figure 3, A). This creates a spontaneous periodic pattern of the two entities (Figure 3, B). Reaction–diffusion systems are involved in development of various tissues and organs. In yeast, Cdc42 regulates polarity through a reaction–diffusion mechanism (Goryachev and Pokhilko, 2008). Cdc42 is activated by its GEF, Cdc24, and this system provides positive feedback due to the recruitment of Cdc24 by activated Cdc42. The reaction components (Cdc42 and Cdc24) diffuse to the cytoplasm of small intracellular pits, which leads to domain competition. This results in a single budding domain in yeast (Kozubowski et al., 2008; Wu et al., 2015).

In Arabidopsis metaxylem cells, ROP-activation/inactivation cycle can generate the ROP11-activated microdomains (Figure 2, C). This would not be achievable without feedback from GEF and slow diffusion of the GEF–ROP complex. Thus, ROP11-activated microdomains are suggested as the first positional cue to directing periodic patterns of cell wall pits in cells (Nagashima et al., 2018). This ability is one of the emerging properties of self-organized systems, where feedback interaction of two elements (here ROP and GEF) is sufficient to create complex/periodic organization. Indeed, and unlike in yeast where there is a single bud site, in metaxylem vessels, the ROP-activation cycle leads to multiple domains that co-exist in the same cells (Figure 2, C). Nagashima et al. (2018) show that level of GEFs and GAPs regulates pitted cell wall patterns in shape and density. They show that GEF4 interacts with ROP11 and induces decreases in ROP11 diffusion. Because GEF4 exists as a dimer, it induces a local recruitment of new ROP11 protein (Figure 3, C; Nagashima et al., 2018). In line with this study, Hwang et al. (2010) described that GAP1 and GDI1 act in maintaining the active ROP1 domain at a proper level and size during rapid continuous pollen growth. Overexpression of GAP1 or GDI1 suppresses the enlargement of the active ROP1 cap induced by ROP1 overexpression. Once ROP1 is inactivated by GAP1, it is probably removed from the pollen tube apex membrane into the cytosol by GDI1. Therefore, the global downregulation of ROP signaling might be linked to the negative feedback needed in the self-organized system (Hwang et al., 2010).

Finally, different from this self-organization principle of reaction–diffusion is the proposed regulation for the root hair initiation domain. In contrast to the multiple cell wall pits of the metaxylem, the positioning and establishment of a single polar root hair at a stereotypical position within the trichoblast plasma membrane is likely to require a mechanism that eliminates randomness during its formation. GEFs have been proposed to play a key role in this non-random polarization. GEF3 mediates the recruitment and temporally controlled ROP activation, followed by later recruitment of GEF4, which gives a higher order of domain organization (Denninger et al., 2019). A similar mechanism was described in Drosophila embryos, where the GEF Dizzy and the heteromeric GEF complex ELMO-Sponge are required for polarization of the GTPase Rap1 (Bonello et al., 2018). What determines the selection of a single ROP activation domain in this case is still an open question. Besides GEFs, other players, such as the membrane lipid composition, are essential to establish the root hair initiation domain (Stanislas et al., 2015). Furthermore, it is likely that GEFs are themselves locally recruited and/or regulated by membrane receptor kinases (Duan et al., 2010; Stanislas and Jaillais, 2019), which could act as positional anchors in connection with the cell wall and extracellular signals (Jaillais and Ott, 2020).

### ROP lipid modifications

**Figure 4:**
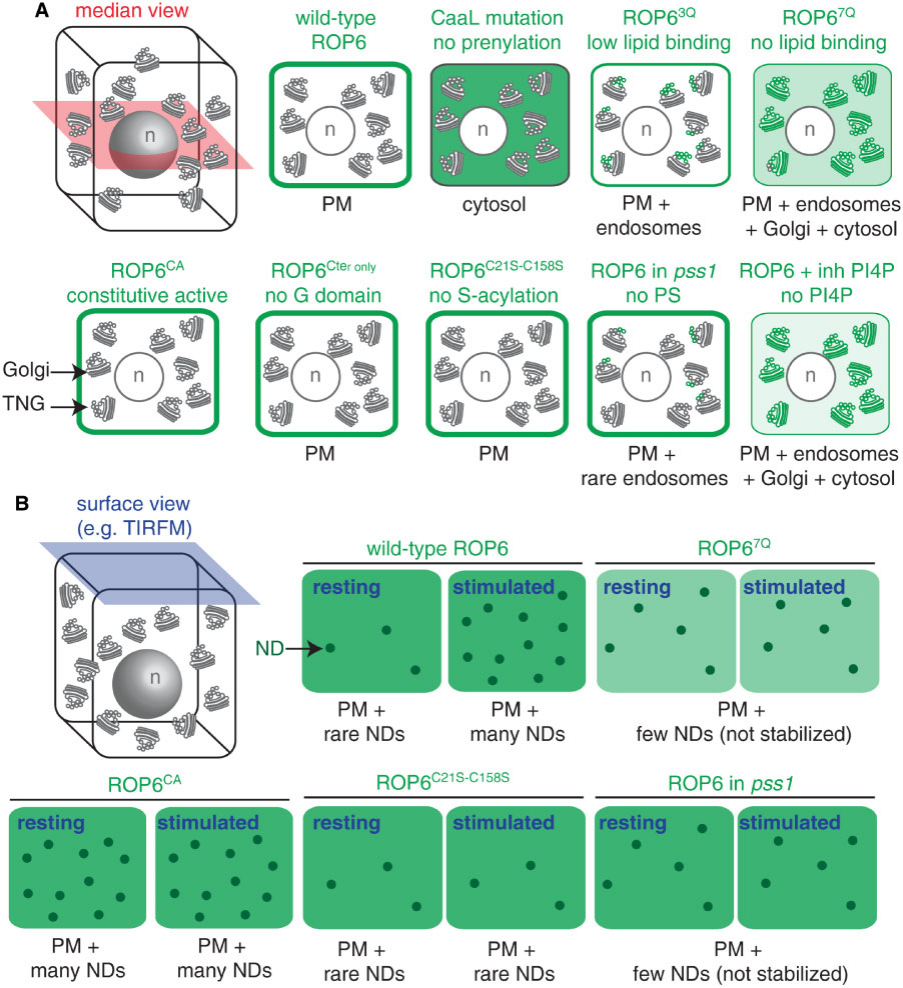
Summary of ROP6 and ROP6 mutant localization depending on the background and activation status. Schematic representation of xFP-ROP6 (where x can be different fluorescent protein) localization as seen by A) confocal microscopy (median view, red), and B) TIRF microscopy (surface view, blue). The ROP6 mutant versions or the background/treatment are indicated on top. In each condition, the localization is indicated in green, with a dark shade of green indicating strong accumulation, while a lighter green indicates weaker accumulation. In B, “Resting” indicates the ROP6 localization in the absence of treatment, while “stimulated” indicates ROP6 localization following auxin treatment or osmotic stress. PM, plasma membrane; ND, nanodomain; inh PI4P, inhibition of PI4P synthesis using a pan-PI4Kinase inhibitor; pss1, phosphatidylserine synthase1; PS, phosphatidylserine; G domain, GTPase domain; n, nucleus. Note that “lipid binding” refers to interaction with anionic lipids via the ROP6 polybasic C-terminal tail.

As mentioned previously, ROP triggers signaling when targeted to the plasma membrane and membrane binding is ensured by posttranslational lipid modifications. Based on their amino acid sequences, ROPs are composed of an N-terminal catalytic GTPase domain where nucleotide and effector binding take place and a C-terminal hypervariable domain, which is responsible, at least in part, for subcellular targeting (Figure 1, A). This C-terminal is composed of Cys motifs and Arg–Lys-rich polybasic regions (Figure 1, C). Arabidopsis ROP1–ROP8 belong to a group of ROPs called Type-I ROPs whose hypervariable region contains a CaaL motif (Figure 1, C). The cysteine can be modified by the isoprenyl lipid geranylgeranyl on the 20C (Feiguelman et al., 2018). The geranylgeranylation of type-I ROPs is required for membrane interaction, and mutations in the CaaL motif that prevent lipid modification, abolish membrane interaction (Figure 4, A;Sorek et al., 2017). Type-II ROPs (ROP9–ROP11 in Arabidopsis) lack the CaaL motif and have instead a GC–CG motif linked by five or six aliphatic residues that undergo *S*-acylation by the C16 palmitate or C18 stearate fatty acids via labile thioester bond (Figure 1, C; Wu et al., 2011). Like for type-I ROPs, lipid modification on the C-terminal tail of type-II ROP is required for membrane targeting (Lavy and Yalovsky, 2006). The adjacent polybasic region is responsible for membrane attachment and interaction with certain membrane lipids (see the section below on lipids).

In addition to their modification in the C-terminal hypervariable region, some ROPs can undergo activation-dependent *S*-acylation in residues inside the G-domain. Indeed, it was found that CA ROP6, but not inactive ROP6, is *S*-acylated in this region with palmitic and stearic acids (Figure 1, A; Sorek et al., 2017). Moreover, auxin treatment, which activates ROP6, also leads to ROP6 *S*-acylation (Pan et al., 2020). Interestingly, ROP6 acylation was linked to membrane partitioning (Sorek et al., 2010). Whereas wild-type ROP6 is present in detergent-resistant membranes and soluble membrane, the CA ROP6-CA mainly accumulates in the detergent-resistant membrane fraction. Point mutations in two cysteines within the GTPase domain that are *S*-acylated abolish the association with the detergent-resistant membrane fraction (Sorek et al., 2010). In addition, ROP6-CA acylation mutant (ROP6-CA C21S/C158S) cannot recapitulate the classical ROP6-CA overexpression phenotype on root hair polar cell growth, endocytosis uptake, and ROS accumulation, suggesting that the lateral segregation of ROP6 within the plasma membrane is important to achieve the ROP6 function (Sorek et al., 2010). The role of acylation on ROP6 nanodomain formation was recently observed directly by a super-resolution microscopy approach. Indeed, whereas ROP6 nanoclustering increases upon hyperosmotic stimulus, the point mutated mEOS2-ROP6 C21S/C158S stays diffusible and does not cluster after cell stimulation (Figure 4, B; Smokvarska et al., 2020). The plasma membrane localization of this acylated mutant was similar to that of the wild-type ROP6 (Figure 4, A). This indicated that proper acylation of ROP6 is dispensable for plasma membrane targeting but essential for its localization in nanodomains. Whereas the mutation of C21A/C158A does not interfere with the GTPase activity of ROP6, these mutations abolished the downstream signaling such as osmotically induced ROS production (Sorek et al., 2010; Smokvarska et al., 2020). ROP2 and ROP10 were also described to undergo *S*-acylation (Lavy and Yalovsky, 2006; Wan et al., 2017). As C158 is conserved among many type-I ROP, *S*-acylation seems to be a general feature of ROP signaling (Sorek et al., 2010, 2011, 2017). How transient *S*-acylation upon activation is regulated and triggers ROP nanodomain formation and maintenance is an outstanding question.

Protein *S*-acyl transferases, or PATs, are integral membrane proteins that catalyze the addition of fatty acyl groups to cysteine residues within proteins, and they have an Asp–His–His–Cys (DHHC) motif (Batistič, 2012). In Arabidopsis, there are 24 PATs. However, only the substrates of PAT4 and PAT10 have been identified (Zhou et al., 2013; Wan et al., 2017). PATs operate by a two-step process. First, the Cys residue of the DHHC motif is auto-acylated by binding an acyl group, such as palmitate or serate. Following this, the acyl group is transferred to a Cys residue in the target protein (Hou et al., 2009; Mitchell et al., 2010; Jennings and Linder, 2012). The cysteine in the DHHC motif is the auto-*S*-acylation site because a mutation in this residue results in loss of auto-acylation of PAT10 (Qi et al., 2013). PAT4 was documented to genetically interact with ROP2, and potentially acylates it. Indeed, in *pat4-2*, ROP2 localization to the plasma membrane is substantially weaker. This is a similar observation as for ROP2^C20S/C157S^, in which the two potential *S*-acylation cysteine residues were mutated into serine (Wan et al., 2017). The mislocalization of ROP2 from the plasma membrane in the absence of PAT4, although substantial, is not drastic, which indicates that PAT4 is not essential for the membrane/cytoplasm partitioning, unlike prenylation, but a putative role in nanodomain formation remains an open question (Chai et al., 2016). In Arabidopsis, 9 out of 24 PATS are located at the plasma membrane, and the rest reside in the endoplasmic reticulum (ER) and the Golgi apparatus (Batistič, 2012). This is different from mammalian and yeast PATs, which are mainly localized to the Golgi (Rocks et al., 2010), and might suggest that regulation of proteins by PATs at the plasma membrane may be more important or could drive membrane subcompartmentalization in plants.

*S*-acylation is a transient lipid modification, suggesting that Acyl Protein Thioesterases (APT) could also participate in the organization of ROP partitioning at the plasma membrane (Tabaczar et al., 2017; Kathayat et al., 2018). APT is able to cleave the thioester linkage between the fatty acid and cysteine sulfhydryl. Unlike in animals and fungi, in plants little is known about the de-*S*-acylation process. Medicago MtAPT1 is the first protein thioesterase with de-*S*-acylation activity that has been found in plants (Duan et al., 2017). More recently, maize ZmB6T1C9 was proposed as *S*-APT, but these data are based solely on structural homology to human acyl-protein thioesterases 2 (APT2; Bürger et al., 2017). To date, there are no reports describing the mechanism of ROP de-*S*-acylation.

### Anionic lipids

#### Electrostatic interactions with anionic lipids are required for ROP plasma membrane targeting

Both type-I and type-II ROPs contain in their hypervariable C-terminal end a polybasic region adjacent to the lipid anchor modification site (Figure 1, C). This region is made of a lysine- and arginine-rich stretch and has a net positive charge that varies from +7 to +9 (Figure 1, C; Platre et al., 2019). Proteins from the Ras/Rho super-family in animals and yeasts also contain a polybasic region, which is known to interact with anionic phospholipids, likely via electrostatic interactions (Heo et al., 2006; Platre et al., 2019). Anionic phospholipids possess a negatively charged head group, and within the endomembrane network, they mostly correspond to phosphatidylinositols and their phosphorylated derivative the phosphoinositides, e.g. phosphatidylinositol-4-phosphate [PI4P] or phosphatidylinositol-4,5-bisphosphate [PI(4,5)P_2_], phosphatidic acid and phosphatidylserine (Noack and Jaillais, 2017). The presence of a polybasic region in ROP C-terminus suggests that they also likely bind to anionic phospholipids. Accordingly, ROP6 interacts with all anionic phospholipids in vitro and these interactions are fully abolished upon the mutation of the ROP6 polybasic region (conversion of seven lysine/arginine residues into neutral glutamine, ROP6^7Q^, which has only two remaining positive charges; Platre et al., 2019). In vivo, a minimal construct consisting of a fusion between the yellow fluorescent protein mCitrine and ROP6 C-terminal tail (polybasic region and CaaL motif) is sufficient to recapitulate ROP6 plasma membrane localization (Figure 4, A; Platre et al., 2019). In addition, the transgenic expression of mCitrine-ROP6^7Q^ shows that these mutations do not fully abolish ROP6 interaction with membranes, but rather impair the nature of the membrane it is targeted to. Indeed, ROP6^7Q^ has a much wider localization than wild-type ROP6, being targeted to the plasma membrane, and also to intracellular compartments (Figure 4, A; Platre et al., 2019). Thus, the interaction between the ROP6 polybasic region and anionic phospholipids likely specifies ROP6 targeting to the plasma membrane, rather than membrane interaction itself. This result is consistent with the notion that ROP6 interacts with anionic lipids through electrostatic interactions. Indeed, the inner leaflet of the plasma membrane is the most electronegative cytosol-facing membrane of plant cells and thus membrane proteins containing polybasic regions with seven-net positive charges or higher tend to be specifically localized to this compartment (Simon et al., 2016). Accordingly, a ROP6^3Q^ mutant, which has six-net remaining positive charges, shows an intermediate localization, with increased plasma membrane targeting compared with ROP6^7Q^, but which is still present in intracellular compartments, in contrast to wild-type ROP6 (Figure 4, A; Platre et al., 2019). The high electrostatic field of the plasma membrane is mainly powered by PI4P, with a more modest contribution of phosphatidic acid and phosphatidylserine (Simon et al., 2016; Platre et al., 2018). Pharmacological inhibition of PI4P synthesis triggers the relocalization of wild-type mCitrine-ROP6 into intracellular compartments, which mimics the localization of mCitrine-ROP6^7Q^ (Figure 4, A; Platre et al., 2019). Thus, the ROP6 polybasic region is required for the specific localization of ROP6 at the plasma membrane, a process that is PI4P dependent.

#### Anionic lipids contribute to ROP polar localization

Given that all ROPs have polybasic regions of similar net positive charges, it is likely that most ROPs will interact with anionic lipids and rely on the plasma membrane electrostatic properties for proper targeting. For example, ROP2 is enriched at the root hair initiation domain together with PI(4,5)P_2_ and a PI4P 5-kinase (PIP5K3; Kusano et al., 2008; Stenzel et al., 2008; Stanislas et al., 2015; Denninger et al., 2019). In addition, type-II ROPs also require their polybasic region for plasma membrane targeting (Lavy and Yalovsky, 2006). In the case of type-II ROPs, a minimal construct containing only the C-terminal tail (polybasic region and GC–CG *S*-acylation motif) is efficiently targeted to the plasma membrane, while deletion of the polybasic region leads to the solubilization of this minimal construct (Lavy and Yalovsky, 2006). Thus, the polybasic regions of type-II ROPs could be required for membrane interaction per se and not only to provide targeting specificity to the plasma membrane, as is the case for the type-I ROPs such as ROP6 (Lavy and Yalovsky, 2006; Platre et al., 2018). In addition, the type-II ROP, ROP10, interacts with PI(3,5)P_2_ and the PI3P 5-Kinase FORMATION OF APLOID AND BINUCLEATE CELLS1 (FAB1; Hirano et al., 2018). ROP10 accumulates in the shank of root hairs together with FAB1 and PI(3,5)P_2_, in a domain complementary to the tip-localized ROP2/4/6, PIP5K3, and PI(4,5)P_2_ (Kusano et al., 2008; Stenzel et al., 2008; Hirano et al., 2018; Stanislas and Jaillais, 2019; Figure 2, A). ROP10 in the shank is required for root hair morphology, likely by regulating microtubule dynamics in this domain. Together this suggests that different ROP isoforms may interact with specific anionic lipids and/or anionic lipid metabolic enzymes to control their polarity.

In addition to ROPs, some ROP regulators also interact with anionic lipids. This is notably the case for the REN GAPs and the scaffolding proteins from the ARO family. REN proteins have a PH domain that binds to phosphoinositides in vitro (Kulich et al., 2020). Furthermore, ARO2 also directly interacts with anionic lipids in vitro, in particular phosphatidylserine and phosphatidic acid. A mutant version of ARO2, in which the lysine and arginine residues in its N-terminal polybasic region were substituted into neutral glycine residues, reduced in vitro lipid binding and failed to localize to the plasma membrane in vivo (Kulich et al., 2020). Of note, a similar situation has been observed in budding yeast as important for Cdc42 polar targeting. Cdc42 directly interacts with anionic lipids via its polybasic C-terminal tail, its GEF Cdc24 via a PH domain, and its scaffolding protein Bem1 via several clusters of basic residues (Meca et al., 2019). Interestingly, Cdc42 and Bem1 localize in plasma membrane nanodomains (Sartorel et al., 2018; Meca et al., 2019), which is also true for both ROPs and AROs (Platre et al., 2019; Kulich et al., 2020; Pan et al., 2020; Smokvarska et al., 2020). However, in yeast, Cdc24 and Bem1 are essentially positive regulators of Cdc42 activation, while REN/ARO are, rather, negative ROP regulators. Although it is so far unknown whether ROP2 colocalizes with REN/ARO in plasma membrane nanodomains, this result nevertheless suggests that nanoclustering could inhibit or terminate ROP signaling, and does not necessarily equate with activation. This will be important to bear in mind in future studies analyzing the function of ROP-containing nanodomains. In any case, scaffolding of Rho and their regulators and multivalent interactions with anionic lipids appear critical to regulate their polar targeting but also their nano-organization at the plasma membrane (as detailed in the next two paragraphs).

#### PI(4,5)P_2_/PIP5K targets NtRac5 in plasma membrane nanodomains in pollen tube

Anionic lipids not only regulate the localization of ROP proteins in polar domains but also their nanoclustering. Indeed, a recent preprint shows that the Arabidopsis AtPIP5K2 localizes in nanodomains at the tip of *N. tabacum* pollen tube (Fratini et al., 2020). AtPIP5K2 directly interacts with the ROP GTPase NtRac5, which also localizes in nanodomains, and together they regulate actin dynamics below the plasma membrane (Ischebeck et al., 2011; Fratini et al., 2020). Using genetically encoded lipid biosensors, Fratini et al. (2020) showed that a PI(4,5)P_2_ biosensor has a dual localization, as it is partly associated with the bulk plasma membrane and partly accumulates in AtPIP5K2-containing nanodomains. In addition, AtPIP5K2 signal extends into the cell cortex below the plasma membrane nanodomains, a zone which is also rich in filamentous actin structures (Fratini et al., 2020). Interestingly, AtPIP5K2 appears to be specifically involved in the biosynthesis of PI(4,5)P_2_ in nanodomains, while PI(4,5)P_2_ present in the bulk plasma membrane is produced by other PIP5K isoforms, including NtPIP5K6. Overexpression of AtPIP5K2 and NtPIP5K6 has distinct pollen tube phenotypes, and by contrast to AtPIP5K2, NtPIP5K6 is important for secretion rather than actin dynamics (Ischebeck et al., 2008, 2010; Fratini et al., 2020). This was elegantly demonstrated using mutant variants of AtPIP5K2 and NtPIP5K6, in which the respective membrane-association domains were swapped. Indeed, an AtPIP5K2 variant with the membrane-association domain of NtPIP5K6 (called 2swap6) localizes to the bulk plasma membrane and its pollen tube overexpression phenotype phenocopies that of NtPIP5K6. By contrast, a NtPIP5K6 variant with the membrane-association domain of AtPIP5K2 (called 6swap2) localizes to nanodomains and phenocopies the phenotypes of AtPIP5K2 overexpression. Together, these results confirm that ROP nanoclustering is a widespread phenomenon happening for many different ROPs, in many different cell types and plant species. This preprint also highlights the exciting possibility that different pools of the same anionic lipids at the plasma membrane may have specific functions in coordinating cellular activities, such as ROP signaling, cytoskeleton dynamics, or secretion (Fratini et al., 2020).

#### Phosphatidylserine stabilizes ROP6 in plasma membrane nanodomains

Like phosphoinositides, phosphatidylserine is also involved in plasma membrane electrostatics, albeit to a lower extent than PI4P and phosphatidic acid (Platre et al., 2018). A mutant in the PHOSPHATIDYLSERINE SYNTHASE1 (PSS1) gene, which completely lacks phosphatidylserine, has little impact on ROP6 plasma membrane targeting (Figure 4, A; Platre et al., 2019). However, the *pss1* mutant is impaired in many ROP6-mediated cellular and developmental responses, including auxin-induced inhibition of endocytosis. This suggests that phosphatidylserine could be required for ROP6 function but not plasma membrane localization. Interestingly, super-resolution single-molecule imaging showed that ROP6 nanoclustering in response to auxin was abolished in the *pss1* mutants and for the mutated ROP6^7Q^ version (Figure 4, B; Platre et al., 2019). Together, these results suggest that ROP6 nanoclustering in response to auxin is required for downstream signaling. As mentioned earlier, ROP6 directly interacts with phosphatidylserine via its polybasic region. Accordingly, phosphatidylserine itself is clustered in nanodomains in the plasma membrane, and these nanodomains colocalize with ROP6-induced nanoclusters following auxin treatment (Platre et al., 2019). In addition, in the context of leaf pavement cells, auxin-induced ROP6 nanoclustering is also dependent upon membrane sterols, which are involved in ROP6 activity in this cell type (Pan et al., 2019).

It is becoming clear that several types of lipids (e.g. sterols, phosphatidylserine, and PI(4,5)P_2_) regulate ROP targeting and stability in plasma membrane nanodomains (Platre et al., 2019; Fratini et al., 2020; Pan et al., 2020). It is possible that these lipids act in concert to regulate ROP nanoclustering or alternatively, that they provide different functional nanodomains that each trigger specific downstream signals. Untangling these possibilities will be a formidable challenge for the coming years.

### Lipid modification and lipid interaction are a multistep process contributing to ROP localization

**Figure 5:**
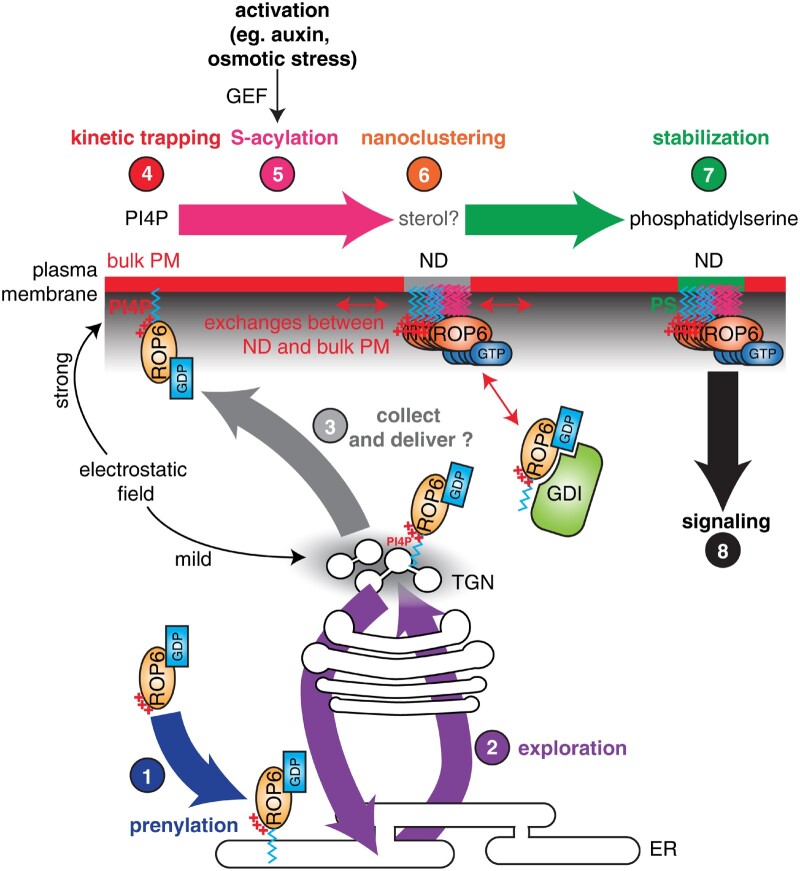
A multistep model for the role of lipid modifications and lipid interactions in the regulation of ROP6 localization and function. Note that this is a hypothetical model with some steps that have not been formally demonstrated (e.g. step 3). The exact order in which the events at the plasma membrane unfold is also currently unknown and this scenario represents one possibility among others. It is, for example, possible that nanoclustering and stabilization are concomitant or that interaction with phosphatidylserine is required upstream of *S*-acylation. PS, phosphatidylserine; ND, nanodomain; red +, positive charge present in ROP polybasic region; blue zig zag line, prenyl chain; magenta zig zag line, acyl chain; GEF, GTPase exchange factor; GDI, guaniside nucleotide dissociation inhibitor.

Taken together, ROP proteins appear to contain multiple and complex localization information. Indeed, we propose that proper ROP membrane targeting is a multistep process involving successive lipid modification and lipid interactions, at least for type-I ROPs (Figure 5). First, type-I ROPs are soluble proteins synthesized in the cytosol. They are then prenylated (geranylgeranylation) at the surface of the ER, which provides hydrophobic anchoring to membrane bilayers. This lipid anchor is sufficient to ensure membrane attachment but on its own, does not provide membrane specificity. Second, in the absence of additional targeting sequences, ROPs can explore many different compartments within the endomembrane network with equal probability (Simon et al., 2016; Platre et al., 2018). This exploration is likely dependent on membrane trafficking as demonstrated by the key role of the TGN-mediated pathway in ROP localization in root hair forming cells and pollen tubes (Stephan et al., 2014; Gendre et al., 2019). Third, the ROP C-terminal polybasic region engages in electrostatic interactions with the negative charges of anionic phospholipids (Platre et al., 2019). After the inner leaflet of the plasma membrane, the TGN is the second most electrostatic cytosolic-facing membrane within plant cells (Platre et al., 2018). This could favor the collection of ROPs at the TGN for their subsequent delivery to the plasma membrane, which could explain, at least in part, the key role of this compartment for ROP localization (Gendre et al., 2019). In addition, the presence of TGN compartments at the tip of root hairs and pollen tubes (Preuss et al., 2004; Szumlanski and Nielsen, 2009; Stephan et al., 2014), close to the plasma membrane tip where ROPs accumulate, also argues in favor of this model. However, note that such a “collect and delivery” system has not been formally demonstrated in plants but has been proposed for human K-Ras (Figure 5; Schmick et al., 2014, 2015). Fourth, the plasma membrane is enriched in anionic lipids compared with the membranes of other compartments (Simon et al., 2016), which allows the plasma membrane to kinetically trap ROPs in a PI4P-dependent manner (Platre et al., 2019). Fifth, upon activation (e.g. auxin, osmotic stress, and genetic CA mutation), ROP6 is *S*-acylated at the plasma membrane (Sorek et al., 2010; Pan et al., 2019). *S*-acylated proteins tend to localize in regions of the membrane that are locally more ordered (Jaillais and Ott, 2020). Upon activation, *S*-acylation could thus act as a trigger to relocalize ROPs into plasma membrane nanodomains, which could contain sterols (Figure 5;Sorek et al., 2010; Pan et al., 2019; Smokvarska et al., 2020). Seventh, these nanodomains are enriched in phosphatidylserine, which directly interacts with ROPs and may stabilize them in these membrane domains (Platre et al., 2019). This model has mostly been investigated with ROP6. However, it is likely that it can be extended to other type-I ROPs, at least to some extent. In the case of ROP6, plasma membrane targeting and nanocluster formation are both required for downstream signaling. An outstanding question in the field is why ROP6 (and possibly other ROPs) need to be localized in nanodomains to be competent for signaling? While this is still largely an open question, in the section below we discuss some hypotheses on the functional importance of ROP nanodomains.

## Effect of nanodomains on signaling parameters

### Specific effector recruitment for a nanodomain specialization in single cells

**Figure 6:**
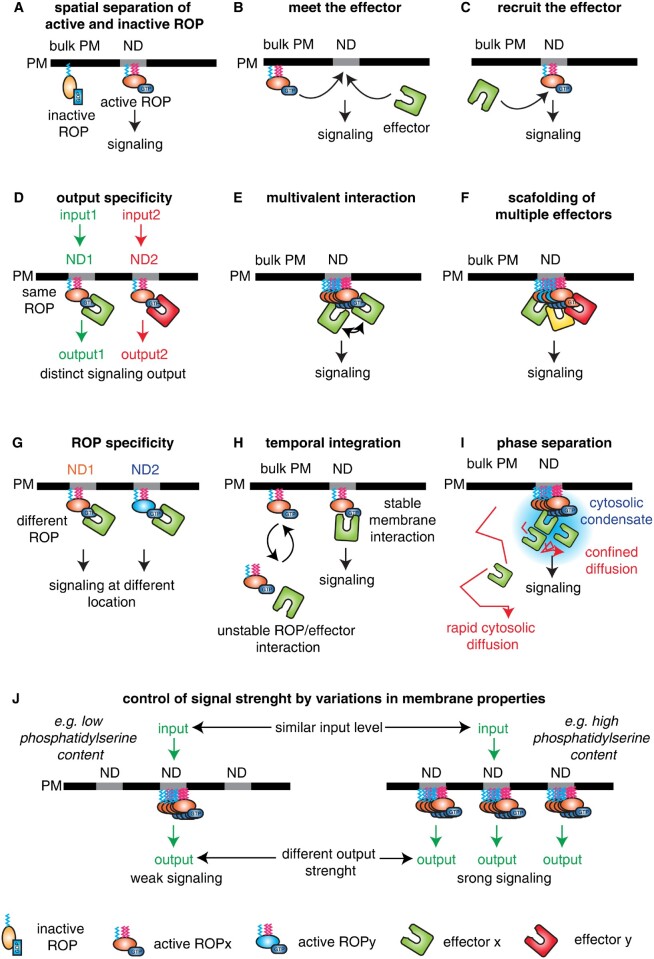
Possible scenarios for the function of nanoclustering in ROP signaling. Note that most of these scenarios have not been formally demonstrated and they remain to be explored. In addition, these scenarios are not mutually exclusive and not exhaustive. A, Nanodomains appear to spatially segregate active (GDP-loaded) and inactive (GTP-loaded) ROPs. However, only a portion (∼30% to 40%) of activated ROPs are localized in nanodomains and this model does not explain by itself why nanodomains are required for downstream signaling. B, Nanodomains could be a favorable membrane environment for ROPs to meet with their effectors, explaining the requirement for nanodomains in signaling. C, ROPs may also recruit their effectors specifically in nanodomains to induce signaling. D, Nanodomains may act as signaling platforms to trigger specific output, as seen in response to auxin treatments or osmotic stress (Platre, 2019; Smokvarska, 2020). E, It is possible that ROP nanodomain formation allows the clustering of downstream effectors, which could induce low energy interactions between them. Such multivalent interaction therefore explains the requirement for nanoclustering in signaling. F, It is possible that multiple ROPs cluster within the same nanodomains, thereby scaffolding several ROP effectors required for signaling. G, It is also possible that multiple ROPs localize to different nanodomains, which could ensure the propagation of specific cytosolic outputs. H, The stabilization of ROPs in nanodomains could increase their dwell time at the plasma membrane, which may be required to stabilize their effector at the cell surface and/or allow them sufficient time to catalyze their reaction. I, The clustering of ROPs in nanodomains may induce the formation of phase-separated cytosolic condensates below the plasma membrane, which could be required for effector function. J, The clustering of ROPs in nanodomains may quantitatively control the strength of the output signal even when the input signal is constant. According to this model, variation in membrane lipids could act like a rheostat that tunes the signaling capacity of cells. Such a model has been proposed for the membrane lipid phosphatidylserine during the ROP6 response to auxin (Platre et al., 2019). PM, plasma membrane, ND, nanodomain. Effectors are represented as a U-shaped protein.

As described throughout this review, there are numerous examples where one ROP isoform is involved in different signaling pathways. For example, ROP6 mediates pavement cell and root hair growth, ROS production, auxin-mediated inhibition of endocytosis, lateral root development, and salicylic acid-regulated pathogen response (Molendijk et al., 2001; Fu et al., 2005, 2009; Sorek et al., 2010; Poraty-Gavra et al., 2013; Venus and Oelmüller, 2013; Wang et al., 2015). These signaling processes often happen in different cell types, and even in different organs, but despite this, studies have also placed ROP6 downstream of different stimuli in the exact same cell type (Platre et al., 2019; Smokvarska et al., 2020). Auxin and osmotic signals act differently on cells by having opposite effects on endocytosis (Chen et al., 2012; Lin et al., 2012; Zwiewka et al., 2015; Martiniere et al., 2019) Whereas ROP6 is mandatory for auxin-mediated endocytosis inhibition, osmotic signals trigger membrane internalization through ROS accumulation (Chen et al., 2012; Lin et al., 2012; Zwiewka et al., 2015; Martiniere et al., 2019). One question that emerges from these findings is how multiple signal stimuli can generate specific responses through the same transducer. In membranes, both osmotic and auxin signaling induce a similar size (around 50 nm) and density of ROP6 nanodomains, but their composition might differ (Platre et al., 2019; Smokvarska et al., 2020). Indeed, Smokvarska et al. (2020) showed that GDP-bound ROP6 colocalized and interacted in nanodomains with the effector protein RBOHD during osmotic stimulation. When cells are exposed to auxin, RBOHD is absent from ROP6-containing nanodomains, suggesting that depending on the upstream signal, the composition of ROP6-containing nanodomains is different (Figure 6, D). Other ROP effectors have a plasma membrane localization that depends on their interaction with ROP (Figure 6, B and C). RIC1 acts as an actin severing protein and needs a fully functional CRIB domain to be targeted to the membrane at the pollen tip growing zone (Zhou et al., 2015). From these different results, we could speculate the existence of signal specific domains where upstream activators such as receptors and/or GEFs coexist before the activation of ROP6 and the recruitment of signal specific effectors (Figure 6, D and G). This constitutes a tempting working model to reconcile the multifaceted roles of ROP in membrane cell signaling.

### Time in nanodomains regulates ROP signaling

Of note, the presence of ROP6 in nanodomains was not fully abolished in the *pss1* mutant, and only the induction of ROP6 nanoclustering after auxin treatment was affected (Platre et al., 2019; Figure 4, B). This provided a unique opportunity to compare the dynamics of ROP6 inside nanodomains in wild-type plants or *pss1* mutants. Fluorescence recovery after photobleaching experiments showed that GFP-ROP6 is highly stable within nanodomains in the wild-type situation (no exchanges between the bleached and unbleached regions for >60 s). By contrast, GFP-ROP6 is not stabilized in nanodomains in *pss1* (exchanges between the bleached and unbleached areas were observed within seconds). This result suggests that ROP6–phosphatidylserine interaction is not required to trigger the relocalization of ROP6 into nanoclusters, but rather to stabilize ROP6 within these structures (Platre et al., 2019). Because ROP6 signaling is compromised when it cannot interact with phosphatidylserine (i.e. *pss1* mutant or expression of ROP6^7Q^), we speculate that ROP6 nanoclustering is required not only for function but also the stabilization of ROP6 in these structures through time. Phosphatidylserine may not be the only element required for the temporal stability of ROP6 nanoclustering, which likely includes the use of multivalent interactions between lipid and protein components within ROP-containing nanodomains (Figure 6, E) and the use of scaffolding proteins such as AROs (Kulich et al., 2020; Figure 6, F).

The importance of phosphatidylserine to stabilize ROP6 in nanoclusters suggests that confining ROPs into nanodomains not only has a spatial function (Figure 6, A–D) but that there is also a temporal aspect of this confinement (Figure 6, H). For example, we can speculate that regrouping ROP6 into nanodomains allows ROP6 to spatially meet its effectors (Figure 6, B, C, and G), but that it also favors their long-lasting interactions, which could be required for downstream signaling (Figure 6, H). Furthermore, it is possible that the enzymatic reactions catalyzed by some of the downstream effector proteins are slow and thus could require long-lasting interactions with activated and clustered ROPs. In addition, increasing the dwell time of ROP6 in nanodomains could impact the local diffusion of cytosolic cortical effector components. In animals, such local slowdown in diffusion has been shown to regulate phase separation of cortical proteins (Case et al., 2019; Huang et al., 2019). The induction of phase-separated condensates in the cytosol, just below plasma membrane nanodomains, could provide local conditions that are favorable for downstream enzymatic reactions (Figure 6, H and I; Jaillais and Ott, 2020). Although this hypothesis remains untested at the moment, it is consistent with the idea that nanoclustering of activated ROP6 is not sufficient to trigger signaling and that ROP6 needs to remain stable in these nanodomains to trigger a downstream effect. Whether membrane-less phase-separated organelles exist below the plasma membrane and in conjunction with plasma membrane nanodomains is an outstanding question for our future understanding of ROP signaling in particular and membrane organization in general.

### Phosphatidylserine acts as a signaling rheostat that modulates ROP signaling output during development

The recruitment of ROP6 in plasma membrane nanodomains in response to auxin scales with the quantity of phosphatidylserine present at the plasma membrane. Indeed, a transgenic Arabidopsis line that overexpresses *PPS1* has a high phosphatidylserine content and an increased recruitment of ROP6 in nanodomains. This induced ROP6 nanoclustering ultimately translates into a hyper-responsive phenotype in term of inhibition of endocytosis by auxin and root gravitropic response (Platre et al., 2019). As such, the *PSS1* overexpressing lines mimic the expression of a constitutive active ROP6. Conversely, plants expressing artificial microRNAs targeting the *PSS1* transcripts have a low phosphatidylserine content, a decreased inhibition of endocytosis by auxin, and slow root gravitropic bending. Thus, the amount of phosphatidylserine at the plasma membrane is rate limiting for ROP6-mediated auxin signaling (Platre et al., 2019; Colin and Jaillais, 2020; Jaillais and Ott, 2020). Interestingly, the localization of phosphatidylserine sensors at the plasma membrane is not uniform in all cells, but rather varies during root development (Platre et al., 2019). Thus, different cells with graded amount of phosphatidylserine will have a distinct auxin response, in term of output strength, at similar auxin concentration (Boutté and Jaillais, 2020; Figure 6, J). These results suggest that ROP nanoclustering is not only required for signaling, but could also tune the strength of signaling outputs at the cell level, with functional consequences at the multicellular scale (Figure 6, J).

## Conclusions and perspectives

Being highly compartmentalized, the plasma membrane can control multiple aspects of cell signaling. This is exemplified by ROP proteins. Indeed, a substantial number of publications have revealed the formation of large domains (larger than 1 µm) in the plant plasma membrane, including at the site for root hair initiation, in the tip of pollen tube or in metaxylem cells (Figure 2). These microdomains determine the recruitment of ROP effector proteins to specific parts of the cell, allowing polar growth or formation of cell wall pits in xylem cells. More recently and thanks to super resolution microscopy, it was revealed that ROP can also form domains at a nanometric scale. These nanodomains are necessary to trigger downstream cell signaling, but how they quantitatively control ROP signaling remains a mostly open question. We proposed a non-exhaustive and non-mutually exclusive list of potential mechanisms by which ROP6 nanoclustering could impact the recruitment, dynamics, and activation of effectors at the membrane (Figure 6).

There are a number of similarities between micro and nanodomains, which suggest that microdomains could be a higher order organization of nanodomains. This idea is supported by the fact that the lipid environment of microdomains and nanodomains presents some similarities. For example, in the root hair initiation domain, ROP2 and ROP6 accumulate in sterol-rich membranes (Stanislas et al., 2015). ROP6 is associated with detergent-resistant membranes, and its clustering and diffusion is sensitive to beta cyclodextrin, a compound that depletes sterol from membranes (Sorek et al., 2010; Pan et al., 2019). The mechanisms underlying the formation of microdomains and nanodomains could also be similar. For instance, in budding yeast, polar localization in microdomains of active Cdc42 is the result of reaction–diffusion processes driven by a small number of molecules that regulate its GTPase cycle (Chant and Herskowitz, 1991; Wedlich-Soldner et al., 2004; Goryachev and Pokhilko, 2008). In plants, a similar hypothesis was proposed for ROP11 microdomains in cell wall pits of metaxylem cells. In addition, mass-conserved activator-substrate models have shown that some parameters of the GTPase cycle, like the saturation point for active Rho, can itself control switching between the unipolar and multipolar domains in cells (Chiou et al., 2018). Therefore, the control of GTPase cycle by GEFs, GAPs, and GDIs might control nanodomain formation in plant cells.

Root hair formation nicely exemplifies the fact that different ROP isoforms could accumulate in distinct microdomains within the same cell. Indeed, whereas ROP2 is localized at the tip of a growing hair, ROP10 is observed in cell shanks (Hirano et al., 2018). This opens questions about whether the same is true for nanodomains: can different ROPs localize to similar or different nanodomains within the same cells or even within the same polar patch (Figure 6, G)? For the moment, only ROP6 and NtRac5 were demonstrated to form nanodomains (Platre et al., 2019; Fratini et al., 2020; Smokvarska et al., 2020), but this property is probably shared with other ROP isoforms. It is possible that ROPs will cluster in the same nanodomains and potentially scaffold multiple effectors to trigger specific outputs (Figure 6, F), or they could accumulate in distinct nanodomains, which could ensure specificity (Figure 6, G). This is a particularly intriguing question since ROPs have only few variations in their amino acid sequences, including conserved acylation sites in the core of the protein and a polybasic sequence at their C-terminus that contains between seven and nine positive charges (Figure 1, C). Only their C-terminal lipidation changes between type-I ROPs that are prenylated and type-II ROPs that are acylated (Figure 1, C). However, work on K-Ras suggests that subtle variations of its C-terminal tail, for example a simple lysine-to-arginine substitution, which does not change its overall net positive charge, may induce interaction specificities for different anionic lipids (Zhou et a., 2017). This suggests that interactions with anionic lipids may not be purely electrostatic and that subtle variations in ROP C-terminal tail may change their anionic lipid specificity. This is an outstanding question to explore in the future (see Outstanding Questions).

**Outstanding questions**

- How, where, and when are ROPs *S*-acylated and de-*S*-acylated?
- What are the similarities and differences between the mechanisms that establish the localization of ROPs in polar domains and nanodomains, and can we extrapolate established concepts of polarity to the nanoscale?
- How does ROP nanoclustering, not just GTP-loading, control effector activation, and to what extent does it contribute to regulation of signaling in a quantitative manner?
- How are ROPs being immobilized at the plasma membrane once activated and what are the impacts of the cell wall, lipids, and membrane receptors in this process?
- Can nanodomains containing different ROPs and/or different effectors co-exist within the same cell, and if yes, what are their impacts in signaling integration and specificity?

## Notes

S.M., Y.J., and A.M. wrote the manuscript.

The author responsible for distribution of materials integral to the findings presented in this article in accordance with the policy described in the Instructions for Authors (https://academic.oup.com/plphys) is: Alexandre Martinière (alexandre.martiniere@cnrs.fr).
